# Percentage on Prescriptions

**Published:** 1847-10

**Authors:** 


*Percentage on Prescriptions.*—A correspondent under the signature of *Pharmacopola*, in the columns of the Annalist, replies to the communication by Medicus, animadverting on some apothecaries, which was copied in our last No. Among other things, he finds fault with Physicians for bargaining to receive a percentage on their prescriptions. We must confess our astonishment at an intimation of the existence of such a custom in this country. We are not prepared to say it does not exist, and our cotemporary, who it is to be presumed is in the way of possessing knowledge on the subject, does not deny its existence. We trust, for the honor of the profession, that if so mercenary and disreputable a custom does prevail in the city of New-York, that the Academy of Medicine will act upon the suggestion of *Pharmacopola*, and pass a decree forbidding it upon pain of forfeiture of fellowship. We should be glad to see the insinuation repudiated, as a calumny on the medical profession of the city of New-York.

Since the foregoing was written, we perceive by the Annalist that this subject has come before the Academy of Medicine, but no definite action has been, as yet, had upon it.

